# [Retracted] Gambogic acid suppresses colon cancer cell activity *in vitro*

**DOI:** 10.3892/etm.2026.13067

**Published:** 2026-01-19

**Authors:** Zailong Zhou, Jian Ma

Exp Ther Med 18:2917–2923, 2019; DOI: 10.3892/etm.2019.7912

Following the publication of this paper, it was drawn to the Editor’s attention by a concerned reader that areas of the flow cytometric (FCM) plots featured in Fig. 2 were strikingly similar to areas within FCM plots ultimately published in other papers in different journals that were written by different authors at different research institutes, some of which had already been submitted for publication prior to the receipt of this article at *Experimental and Therapeutic Medicine*, and a few of which have since been retracted.

Owing to the fact that the contentious data in the above article were found to be strikingly similar to data that have been published elsewhere in other papers in different journals, the Editor of *Experimental and Therapeutic Medicine* has decided that this paper should be retracted from the Journal. The authors were asked for an explanation to account for these concerns, but the Editorial Office did not receive a reply. The Editor apologizes to the readership for any inconvenience caused.

